# Exercise and manual physiotherapy arthritis research trial (EMPART): a multicentre randomised controlled trial

**DOI:** 10.1186/1471-2474-10-9

**Published:** 2009-01-19

**Authors:** Helen P French, Tara Cusack, Aisling Brennan, Breon White, Clare Gilsenan, Martina Fitzpatrick, Paul O'Connell, David Kane, Oliver FitzGerald, Geraldine M McCarthy

**Affiliations:** 1 School of Physiotherapy, Royal College of Surgeons in Ireland, 123 St Stephen's Green, Dublin 2, Ireland; 2 School of Physiotherapy and Performance Science, University College Dublin, Belfield, Dublin 4, Ireland; 3 Adelaide, Meath Hospital Dublin (incorporating the National Children's Hospital), Tallaght, Dublin 24, Ireland; 4 Mater Misericordiae University Hospital, Eccles St, Dublin 7, Ireland; 5 Beaumont Hospital, Dublin 9, Ireland; 6 St Vincent's University Hospital, Elm Park, Dublin 4, Ireland

## Abstract

**Background:**

Osteoarthritis (OA) of the hip is a major cause of functional disability and reduced quality of life. Management options aim to reduce pain and improve or maintain physical functioning. Current evidence indicates that therapeutic exercise has a beneficial but short-term effect on pain and disability, with poor long-term benefit. The optimal content, duration and type of exercise are yet to be ascertained. There has been little scientific investigation into the effectiveness of manual therapy in hip OA. Only one randomized controlled trial (RCT) found greater improvements in patient-perceived improvement and physical function with manual therapy, compared to exercise therapy.

**Methods and design:**

An assessor-blind multicentre RCT will be undertaken to compare the effect of a combination of manual therapy and exercise therapy, exercise therapy only, and a waiting-list control on physical function in hip OA. One hundred and fifty people with a diagnosis of hip OA will be recruited and randomly allocated to one of 3 groups: exercise therapy, exercise therapy with manual therapy and a waiting-list control. Subjects in the intervention groups will attend physiotherapy for 6–8 sessions over 8 weeks. Those in the control group will remain on the waiting list until after this time and will then be re-randomised to one of the two intervention groups. Outcome measures will include physical function (WOMAC), pain severity (numerical rating scale), patient perceived change (7-point Likert scale), quality of life (SF-36), mood (hospital anxiety and depression scale), patient satisfaction, physical activity (IPAQ) and physical measures of range of motion, 50-foot walk and repeated sit-to stand tests.

**Discussion:**

This RCT will compare the effectiveness of the addition of manual therapy to exercise therapy to exercise therapy only and a waiting-list control in hip OA. A high quality methodology will be used in keeping with CONSORT guidelines. The results will contribute to the evidence base regarding the clinical efficacy for physiotherapy interventions in hip OA.

**Trial Registration:**

Number: NCT00709566

## Background

Osteoarthritis can be defined as a group of overlapping distinct diseases, which may have different aetiologies but with similar biologic, morphologic and clinical outcomes. The articular cartilage degenerates with the development of fibrillation and fissures, and full thickness loss of the joint surface' [1]. It is estimated that by 2030 the proportion of people with OA will have risen from 20% to 30% in those aged 60 years or over [2]. Increasing life expectancy, decreasing physical activity, and increasing body weight are all considered as underlying factors. OA is the most common form of arthritis and is associated with a considerable cost to the individual and to society. A World Health Organisation report identified OA as the 8^th ^leading cause of non-fatal burden in the world in 2000, accounting for 2.6% of total years lost due to disability [3]. It is commonly associated with other medical conditions such as cardiovascular disease, respiratory disease and diabetes [4,5]. Prevalence of hip OA varies between 1.4% and 3.5% based on radiographic definitions [6], and between 0.7 and 4.4% for symptomatic hip OA [7]. Physical function and physical activity levels are reduced in hip OA and consequently this can impact on quality of life [8-10] and has associated psychosocial impact such as depression [11-13].

To date, there is no cure for OA, so the principles of management are to control pain, improve function and reduce disability [14,15]. Contemporary thinking is that non-pharmacological measures such as patient education, weight loss, physiotherapy, occupational therapy, and exercise should be tried first, with pharmacological intervention used as an adjunct [16]. Physiotherapy is the most common non-pharmacological intervention prescribed for OA [17] and aims to reduce pain and restore or maintain optimum physical functioning [18].

Physiotherapy can comprise a number of different interventions, including electrotherapy, massage and the provision of orthotics, but a questionnaire survey of Irish physiotherapists in public and private settings identified that the most common interventions used by physiotherapists to manage hip OA were exercise therapy (100%), education (99%) and manual therapy (96%) [19].

### Evidence for Exercise Therapy

Therapeutic exercise has been recommended as a key component of the management of hip OA in a number of clinical guidelines [20-24]. It can comprise joint-specific exercise for range of motion, strengthening of muscles around the hip and general aerobic conditioning. It can take place on land or in water (hydrotherapy) and can be done in a supervised setting or as a home-based self-directed programme [25]. A number of studies have evaluated the effect of exercise therapy in hip OA. Different forms of exercise therapy have been investigated such as hydrotherapy [26-30] and strengthening [29,31-34]. Exercise has also been used as a control intervention with the provision of advice/education and a programme of home exercises [28,35].

Although a number of systematic reviews have been undertaken evaluating the effectiveness of exercise therapy in OA, most of these have included knee and hip OA studies [36-39], with just one focussing specifically on hip OA [40]. This review, based on nine RCTs, found that therapeutic exercise, with a strengthening component, was an efficacious treatment for pain in hip OA. To date, the benefits of exercise for improving pain and function in hip OA appear to be short-term only [32,34,38] However, many of the studies undertaken have combined hip and knee OA patients [29,30,32,41] thereby limiting the value of the results. These studies were not powered sufficiently to observe the effect of exercise on hip OA only. Secondly, the same exercise regime was used by patients with hip and knee OA, thus the specificity of the exercise regime to hip OA is questionable. Certain flaws existed in many of the studies such as inadequate sample size [29,34], lack of intention-to-treat analysis [41,42] and lack of blinding of outcome assessors [29,42]. Due to the nature of exercise based interventions, blinding of the subjects and treating therapists was not possible in any of the studies [43].

### Evidence for Manual Therapy

Manual therapy is a commonly used intervention in the management of musculoskeletal dysfunction in physiotherapy. It can be defined as 'a clinical approach involving specific hands-on techniques including, but not limited to specific hands-on mobilization, that are used by the physical therapist to diagnose and treat soft tissues and joint structures for the purpose of modulating pain; increasing range of motion; reducing or eliminating soft tissue inflammation; inducing relaxation; improving repair, extensibility or stability of contractile or non-contractile tissue; facilitating movement and improving function' [44] (pg 180). Manual therapy comprises manipulation and mobilisation techniques. Manipulation consists of forceful, high velocity thrusts, whilst mobilisations are less vigorous techniques which are used more often than manipulation for peripheral joint pain and stiffness [17]. Despite its widespread use clinically, there is little scientific evidence to substantiate the effectiveness of manual therapy in reducing pain or improving function in OA [45]. Specifically in hip OA, there has been considerably less research into the effectiveness of manual therapy compared with exercise therapy.

To these authors' knowledge, only one clinical trial has been conducted evaluating manual therapy in hip OA [33]. Manual therapy, which encompassed manipulation techniques and stretching, was compared head to head against exercise therapy. Results at 5 weeks demonstrated 81% improvement in the manual therapy group and 50% in the exercise therapy group. This was based on a 6-point Likert scale of patient perceived general improvement. These data may not be relevant to clinical practice in Ireland for two reasons. Firstly, in Ireland, therapists more commonly use mobilization techniques developed by Mulligan [46] and Maitland [47] rather than manipulation [19]. Secondly, current clinical practice shows that manual therapy is frequently used in combination with other interventions such as exercise therapy [19] and there is a need to conduct randomized controlled trials that are transferable to clinical practice [48]. To date, no known studies have evaluated the combined effect of manual therapy and exercise in hip OA. Recently published UK clinical guidelines on the care and management of osteoarthritis in adults recommended manual therapy as an adjunctive treatment to core treatments of exercise and education in OA, particularly hip OA [20].

### Predictors of response to treatment

Treatment effectiveness can be improved by matching treatment to patient characteristics to determine what works best for whom. Few studies have evaluated predictors of response to physiotherapy in hip OA. Hoeksma et al [49] found that patients with mild/moderate radiographic changes who received manual therapy had significantly better range of motion outcomes than those with severe changes. Manual therapy had no differential effect on subgroups of patients defined by baseline pain or hip function. This secondary analysis did not evaluate the outcome of the exercise intervention on different subgroups. Female gender, low co-morbidity, absence of depressive symptoms and a history of receiving complementary medicine in the previous 12 months were the most stable predictors of outcome in a non-randomised study of inpatient rehabilitation for hip or knee OA [50]. In this proposed study, patients of varying severity will be included and it is possible that treatment effects may be different in groups of patients with different characteristics, such as x-ray severity and symptomatic severity.

In summary, a multi-centre randomised controlled trial that compares the clinical effectiveness of a combination of manual therapy and exercise therapy, exercise therapy only and a waiting-list control will be conducted. This study also will examine if any clinical baseline features predict response to treatment as a secondary analysis.

## Methods

### Aim

The primary aim of this study is to compare the effect of a combination of manual therapy and exercise therapy, exercise therapy only and a waiting-list control on physical function in hip OA.

A secondary aim of the study will be to investigate the effect of baseline variables such as age, gender, body mass index, disease severity, baseline pain, physical function, mood and co-morbidities on treatment outcome.

### Study Design

A multi-centre assessor blind RCT that evaluates the clinical effectiveness of two physiotherapy interventions for patients with osteoarthritis of the hip will be conducted. The methodology will follow CONSORT (Consolidation of Standards of Reporting Trials) guidelines [51,52].

### Ethics

Ethical approval for this study has been granted by the research ethics committees of the four participating hospitals: St James' Hospital/Adelaide Meath Hospital Dublin, incorporating the National Children's Hospital research ethics committee, Beaumont hospital ethics medical research committee, St Vincent's Healthcare Group Ltd ethics and medical research committee and Mater Misericordiae University Hospital research ethics committee.

### Participants

Potential participants will be recruited from the physiotherapy waiting lists in the four participating hospitals if they meet the inclusion criteria as shown in Table 1.

**Table 1:** Inclusion/Exclusion Criteria

**Inclusion Criteria**
1. Subjective complaint of hip pain with either hip internal rotation < 15° and hip flexion <115° or
2. ≥15° hip internal rotation and pain on hip internal rotation, morning stiffness less than or equal to 60 minutes, age > 50 years. (*American College Rheumatology Clinical criteria for the diagnosis of hip osteoarthritis*) [53]
3. Age 40–80 years except in 2 above (age >50 years).
4. Radiological evidence of osteoarthritis (2 of the following 3 criteria): osteophytes, joint space narrowing, ESR<20 mm/hr. (*American College of Rheumatology Criteria for the Classification and Reporting of Osteoarthritis of the Hip*) [53].
**Exclusion Criteria**
1. Previous hip arthroplasty, history of congenital/adolescent hip disease
2. Clinical signs of lumbar spine disease
3. Physiotherapy in previous 6 months
4. Pregnancy
5. Hip fracture
6. Contraindications to exercise therapy (unstable angina/blood pressure, myocardial infarction in previous three months, cardiomyopathy, uncontrolled metabolic disease, recent ECG changes, advanced COPD, third degree heart block)[54]
7. On waiting list for joint replacement within the next 27 weeks
8. Rheumatic diseases e.g. Rheumatoid Arthritis, Ankylosing Spondylitis, etc.
9. Intra-articular hip corticosteroid injection in previous 30 days
10. Insufficient English language to complete questionnaires

### Recruitment Procedure

All patients with a diagnosis of hip OA referred for physiotherapy from rheumatologists, GPs, orthopaedic consultants and other hospital consultants will be considered for inclusion in the RCT. Four physiotherapy departments in large acute hospitals in the Dublin area will be used: Beaumont Hospital, St Vincent's University Hospital, Adelaide Meath Hospital Dublin, incorporating the National Children's Hospital and Mater Misericordiae University Hospital.

Patients will be initially contacted by letter to explain the purpose of the study. A copy of the participant information leaflet will be enclosed. An initial telephone screening interview will be conducted to screen for major exclusion criteria and to provide the participant with more detailed information about the study. Potentially suitable participants will then be invited to attend an appointment with the blinded outcome assessor (HPF) who is a qualified physiotherapist. At this time, a comprehensive musculoskeletal examination will be undertaken to verify the volunteer's suitability for inclusion, based on the inclusion criteria outlined in Table 1. If eligibility is confirmed, patients will complete the consent form.

### Randomisation

Two computer generated randomisation lists have been drawn up by a statistician who is independent of the study. The first list will be used to randomize participants into one of the three arms of the trial. The second list will be used to re-randomise those subjects in the control group into one of the two intervention groups after the 9 week follow-up assessment. Both lists will be maintained by a member of the research team (TC) who will not be involved in the assessment or treatment of the participants.

Once informed consent has been obtained and baseline outcome measures have been completed, each participant will be randomly allocated to one of 3 groups

a. Exercise therapy

b. Exercise therapy and Manual Therapy

c. Control group (Waiting List)

Simple randomisation will be conducted. Group allocation will be communicated to the treating therapists in each treatment centre by the independent randomiser.

### Interventions

Interventions will be administered by senior chartered physiotherapists in the four participating hospitals. All treating therapists will have attended two training sessions which will focus on the exercise and manual therapy interventions to ensure a standardised approach to treatment across the four trial centres.

The three components of the trial are as follows:

### Exercise therapy

Participants will attend six to eight 30 minute physiotherapy sessions over eight weeks. Each treatment will be administered on a one to one basis. The exercise intervention will incorporate flexibility and strengthening exercise using a semi-structured protocol, which provides guidance on exercise prescription and progression, but can be tailored to individual patient physical assessment findings. The content of the protocol is based on the findings of a questionnaire survey of the practice of Irish physiotherapists in the management of hip OA [19] and clinical guidelines for the management of hip and knee OA [21,24,54,55]. The focus of the strengthening programme is based on low load exercise, commencing in non weight bearing positions and progressing to functional positions [54]. The key target muscles are the gluteal muscles which are commonly atrophied in hip OA [56-58]. A range of exercises to address the various degrees of severity of hip OA will be available. Patients will also undertake a daily home exercise programme to supplement the clinic based treatment. They will complete an exercise log which will be reviewed by the treating therapist at each clinic attendance.

Each subject will be encouraged to undertake some form of aerobic exercise (such as walking, cycling, swimming) over the intervention period. They will be given written and verbal information on principles of aerobic conditioning such as pacing, gradually progressing intensity and time of exercise, doing 'little and often' and incorporating exercise into daily life. They will also be advised of cardiovascular warning symptoms/signs to be aware of, which may require review by a medical practitioner. Participants will be encouraged to achieve recommended aerobic exercise target of at least 30 minutes, five days a week [59].

### Exercise Therapy and Manual Therapy

Participants in this group will attend six to eight 45 minute sessions of physiotherapy over an eight week period. This will include 30 minutes of exercise therapy and 15 minutes of manual therapy. A choice of manual therapy techniques can be used which will be based on the pain/stiffness relationship as well as the movement restrictions of the affected hip. These will be ascertained by the blinded outcome assessor to ensure standardization and will be communicated to the treating therapists on a referral form. Treating therapists may choose from a list of manual therapy techniques based on Maitland [47], Mulligan [46], Cyriax [60] and other mobilization techniques such as muscle energy techniques [61] and proprioceptive neuromuscular facilitation (PNF)[62].

### Control Group (Waiting List Control)

Participants in the control group will continue to wait on the physiotherapy waiting list for an eight week period. On the 9^th ^week, they will complete a follow-up assessment with the blinded outcome assessor. They will then be re-randomised into one of the two intervention groups.

All groups will receive standardized written information on hip OA as recommended by clinical guidelines [22] including a standardised educational booklet entitled 'Osteoarthritis: helping you to understand osteoarthritis' and 'Osteoarthritis-Questions and Answers' published by Arthritis Ireland. This will ensure that education is not a confounding variable in the study.

All non-consenting and excluded participants will be treated as usual by the physiotherapy department of each trial centre. Participants will be asked to avoid all other forms of intervention for the duration of the RCT, apart from routine doctor care and analgesics. Participants with bilateral hip OA will receive treatment for both hips, but outcomes will be assessed on the worst affected hip only.

### Outcome Measures

**Figure 1:**
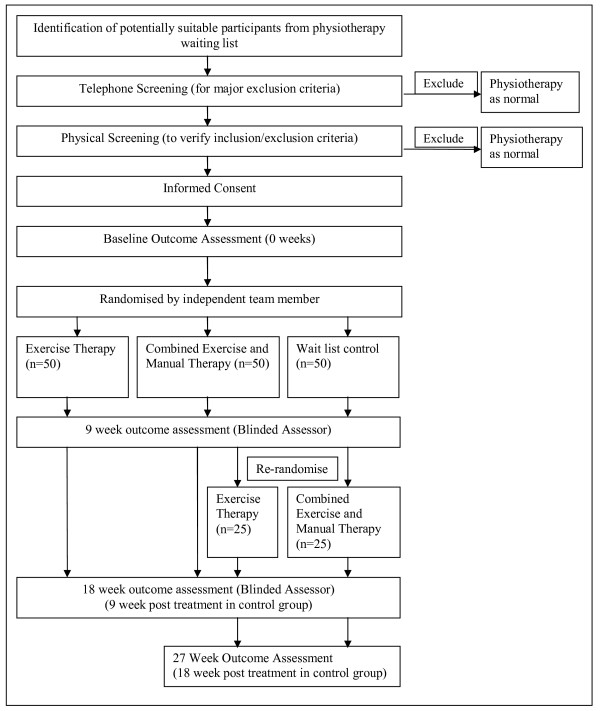
**Flow of participants through the RCT**.

Patients will be assessed at baseline, 9 weeks (end of treatment for the intervention groups) and 18 weeks (end of treatment for the control group) by the blinded assessor in the physiotherapy department of each hospital. Subjects in the control group will have an additional follow-up at 27 weeks (18 weeks post treatment) (Fig 1). Subjects and treating therapists cannot be blinded to treatment allocation due to the nature of the interventions.

Standardised instruments with proven validity and reliability and in keeping with the core set of outcome measures of pain, physical function and global assessment as recommended by OMERACT III (Outcome measures in Rheumatology Group) [63] for use in osteoarthritis will be used. All outcomes will be measured at baseline, 9, and 18 weeks (and 27 weeks for the control group), unless otherwise stated.

The primary outcome measure will be:

### WOMAC (Western Ontario and McMaster Universities) Osteoarthritis Index Physical Function Subscale

The WOMAC is a multidimensional, self-administered health status instrument. It is available on both a 5-point Likert scale and a 100 mm visual analogue scale (VAS). In this study, the Likert version will be used. WOMAC consists of 24 questions regarding pain (scored 0–20), stiffness (scored 0–8) and physical function (scored 0–68). It has been shown to be a reliable, valid and responsive multidimensional outcome measure in patients with osteoarthritis of the hip or knee [64] and has been found to demonstrate superior responsiveness to the Lequesne Algofunctional Index [65]. Minimal perceptible clinical improvement has been found to be 9.3 mm on the physical function subscale on the WOMAC 100 mm normalised VAS [66].

Secondary Outcome Measures include:

### Global Assessment of Change (GAC)

Patient global assessment is one of the core domains which was recommended by OMERACT for evaluation in phase III trials in OA [63]. GAC comprises a single question which asks participants about the degree of change since their previous assessment and is therefore retrospective or transitional in nature. It functions as an external criterion to differentiate between changed and unchanged patients at the end of the study. It is subject to recall bias and can be heavily influenced by current status and patient satisfaction [67]. A 7-point Likert scale will be used ranging from 'very much worse' to 'very much better', with 'no change' located in the middle. The minimal clinically important difference of an average change of 0.5 on a 7-point scale has been found [68,69]. This will be measured at 9 and 18 weeks (and 27 weeks for the control group).

### Numerical Rating Scale (NRS) for Pain Severity

This is an 11-point scale that measures pain severity. Subjects will be asked to rate pain severity in the previous 24 hours with activity, at night and at rest. It is reliable, valid and preferable to a visual analogue scale in pain measurement, especially in an older population [70,71]. A change of approximately 2 points or 30% is considered to be clinically important [72].

### Short Form-36

This is a widely used well validated self-completed questionnaire that measures perceived health status and quality of life. It consists of eight multi-item scales measuring physical functioning (PF), bodily pain (BP), role limitations due to physical health problems (RP), general health (GH), vitality (V), social functioning (SF), role limitations due to emotional problems (RE) and mental health (MH)[73]. The scores on all subscales range from 0–100 with higher scores indicating better health status. A physical and mental health score can be derived from the items. The SF-36 has been used in other rehabilitation studies of hip OA [30,33,74].

### Hospital Anxiety and Depression Scale (HADS)

This self-report questionnaire measures mood disorder and is validated for use as a screening tool in the general population [75]. It contains 14 items, 7 relating to anxiety and 7 relating to depression, which are scored separately. A score of 0–7 indicates no anxiety or depression, 8–10 is borderline and 11–21 indicates the presence of anxiety or depression [76]. Due to the strong links between OA and psychological wellbeing [77,78], this outcome will be used to identify if anxiety or depression are confounders to treatment response and predictors of treatment outcome.

### International Physical Activity Questionnaire (short version)

This is a self-report questionnaire which asks participants to recall the amount of physical activity undertaken over the previous 7 days. It has been extensively validated across 12 countries for the measurement of physical activity in adults age 18–69 years [79].

### Physiotherapy Out-Patient Survey for patient satisfaction

This validated self-report questionnaire was specifically developed to measure patient satisfaction with physiotherapy in an outpatient setting. It measures dimensions of expectation of physiotherapy, communication, perceptions of the therapist, organization of treatment sessions, content of treatment session and outcome of treatment [80]. It will be completed at the 18 week follow-up assessment only.

### Pain Medication Usage (Pain Diary and Medication Quantification Scale (MQS))

Medication usage is a central management strategy in OA [15,20] and it is therefore important to measure the potential change in this usage over the course of the RCT. The MQS is an established, partially validated instrument that quantifies pain medication usage over a period of time. Each pain-related medication that participants are taking is given a score that is derived by multiplying the detriment weight of the drug and the relative daily dosage [81]. A single score is calculated that can be compared at different points in time and includes weighting for detrimental effects of the medication [82]. The daily dosage of pain medications will be measured using a medication diary which the participants will record over a 7 day period.

### Hip Range of Motion

Loss of range of motion (ROM) is a common clinical finding in hip OA and is associated with pain and disability [83]. Active range of motion will be measured on the affected hip. The movements of flexion, abduction, medial rotation and Thomas test will be measured using a universal goniometer. High test-retest reliability values for flexion, abduction and medial rotation have been reported (0.82, 0.86 and 0.90 respectively using the intraclass correlation co-efficient (ICC)) in subjects with hip OA [84]. A combined movement of flexion, abduction and external rotation (FABER) or the Patrick test will be measured using a measuring tape. This has been found to have a high test-retest reliability (ICC = 0.93) in healthy college-aged men [85] and a high correlation with radiographic changes in hip OA (Pearson's r = 0.54, p < 0.01). It is recommended for assessment of secondary endpoints in clinical trials [86].

### Physical Performance

Physical performance measures should be used in combination with self-report measures in the assessment of physical function [87]. In this study, two measures of physical performance will be used.

#### Repeated Sit to stand

This is one of a battery of measures used by Simmonds et al [88] to measure physical performance in subjects with chronic disorders. The task uses a standard armchair and requires the patient to stand from a sitting position and, as fast as possible, to return to the seated position 5 times. The task is repeated after a brief pause and the average time of 2 tasks is the resulting score. The average of 2 trials is necessary for acceptable reliability [89]. It has demonstrated excellent test-retest reliability (ICC = 0.83) and day to day retest reliability (ICC = 0.89) in 48 healthy pain-free subjects [89]. It has been used as an outcome measure in other OA studies [42,90].

#### 50 foot walk test

The 50 foot walk test is also one of the battery of physical performance tests used by Simmonds et al [88]. Subjects are asked to walk at their fastest speed along a premarked walk way of 25 feet, turn and return to the starting point. It has demonstrated test-retest reliability of ICC = 0.91 and day to day retest reliability of ICC = 0.65 in control subjects and ICC of 0.99 and 0.80 in low back pain subjects [89]. It has previously been used in a exercise-based osteoarthritis trials [91-93].

### Sample Size Considerations

The primary outcome measure-WOMAC physical function subscale- has been used to estimate the sample size required. The minimum clinically important difference (MCID) has been ascertained [66]. Using an MCID of 5.4 (SD = 11) on WOMAC Likert scale, with a significance level of 0.05 (2 tailed) and a power of 80%, it is estimated that 67 patients are required per group. To allow for 10% attrition at the 9 week follow-up, it is estimated that 74 patients will be required per group. However, as the waiting list control group will be re-randomised into 2 treatment groups after the 9 week follow-up, 50 patients per group will be required to give a total sample size of 150 patients.

### Statistical Analyses

These will be blinded and will be performed in consultation with a statistician. Baseline and demographic data will be presented using descriptive statistics. Differences from baseline will be calculated for all primary and secondary outcomes. Mean differences, standard deviations and 95% confidence intervals will be calculated for all continuous outcomes. A longitudinal repeated measures design will be used to assess change from baseline and to make between group comparisons for the continuous outcome measures. Comparisons will be made between the 3 groups at the 9 week period. Participants in the control group will be analysed according to the intervention received after the 9 week follow-up and comparisons will be made between the 2 intervention groups at 18 weeks (both with and without the re-randomised control group subjects at the 18 week period). This will be done on an 'intention to treat' basis. Participants will be analysed in the intervention groups to which they were originally assigned and will include withdrawels and patients not treated by the assigned intervention. Missing data will be replaced using the last observation carried forward. Per-protocol analyses will also be performed by excluding patients with deviations from the treatment protocol.

At the 9 week and 18 week time points, continuous variables will be analysed using a mixed ANOVA model (with treatment as the between group factor and time as the repeated factor), or the non-parametric equivalent if non-normal distributions apply. Where significant differences occur, post-hoc comparisons will be conducted using Tukey's multiple comparison procedure. Differences in nominal/ordinal data will be analysed using the χ^2 ^test. A significance level of 0.05 will be set for any inferential statistics conducted.

Secondary analysis will be undertaken to assess the predictors of outcome at primary endpoint (9 weeks post treatment) in both intervention groups, using multiple regression analyses. A number of predictor variables such as age, gender, x-ray severity, baseline pain, physical function, mood, co-morbidities and treatment expectations will be measured. Analyses will be performed separately for exercise therapy, and combined exercise and manual therapy. Response variables will be change in WOMAC physical function score pre and post intervention (MCID> 5.4) and Global Assessment of Change. All predictors will be checked for collinearity. Univariate logistic regression will be performed on all potential predictors and those associated with the outcomes will be entered into the multivariate logistic regression model and backward regression will be performed. Variables with the lowest predictive value will be removed from the model if p > 0.05. Odds ratios and 95% confidence intervals will be calculated for all final predictors.

## Discussion

This paper outlines the protocol for a multicentre RCT that will compare the effect of a combination of manual therapy and exercise therapy, exercise therapy only and a waiting-list control on physical function in osteoarthritis of the hip. A multicentre approach was chosen to maximize the sample size and to improve generalisability of the results. This study is in keeping with Meinert's definition of multi-centre, where the data are acquired from two or more settings that are organizationally independent, a common intervention and data-collection protocol are used, and data management and analysis are centralized [94]. Although the setting of the four centres is similar, there may be differences in the demographic and socioeconomic profile between the sites, which are located in different geographical areas within an urban setting.

A number of methodological elements have been included in the trial to minimize bias such as power calculation, randomization, concealed allocation, blinded outcome assessment and intention-to-treat analysis. The reporting of the study will be in keeping with CONSORT guidelines [51], including recent recommendations for the reporting of non-pharmacological studies [52].

A distinctive feature of the study is the inclusion of a waiting-list control group. This is a variation of a cross-over trial, where the participants in the control group are crossed over to one of the intervention groups after the 9 week follow-up assessment. This design was chosen, as one of the inclusion criteria for participation in the trial was that all subjects must have been referred for physiotherapy, and consequently must receive treatment. As the waiting times for physiotherapy at the four participating sites can vary between 6–12 weeks, this waiting-list control group will not be unduly compromised in their waiting times for treatment. A true control group can result in higher dropouts as patient expectations are not met and implementing a cross-over design may help to reduce dropout rates in studies investigating chronic diseases such as arthritis [95]. The decision to include a control group was made because, although exercise therapy has been shown to have a positive short term effect on pain and function in hip OA, the specific exercise protocol in this study has not previously been investigated. Both exercise and manual therapy protocols are based on current clinical practice identified through questionnaire survey [19] and therefore should be easily implemented in a clinical setting. The waiting-list control group will improve the scientific rigour of the study and allows more definite conclusions to be made regarding the effect of the two interventions under investigation. The control group patients will be re-randomised into one of the two intervention groups after the 9 week follow-up. A similar design was used by Fransen et al [91,96]. Statistical analyses will be conducted on the two intervention groups, with and without the re-randomised control group subjects, to assess the effect of the 9 week waiting period on outcomes.

A combination of self-report and physical outcome measures will be used, all of which are easy to administer in a standardized manner across the four sites.

A secondary aim of this trial is to ascertain the effect of a number of baseline variables on treatment outcome of the two interventions under investigation. Effect of treatment may be different for patients with specific characteristics. Identification of factors that affect treatment response is increasingly being recognized as an important aspect of physiotherapy management as it may facilitate the development of more tailored rehabilitation regimes [97-99]. This is particularly relevant in OA which is described as a heterogenous condition [100]. The EULAR evidence-based recommendations for the management of hip OA identified that clinical predictors of response to pharmacological and non-pharmacological interventions for hip OA was one of 10 future research topics which should be undertaken [22].

In summary, this multi centre RCT will be undertaken to compare the effect of a combination of manual therapy and exercise therapy, exercise therapy only, and a waiting-list control on physical function in hip OA. High quality methodology will be used in keeping with CONSORT guidelines. The results of the study should inform clinical practice and add to the evidence base for physiotherapy-based interventions in hip OA.

## Competing interests

The authors declare that they have no competing interests.

## Authors' contributions

All authors were involved in the design of the study. GMcC and TC and will act as supervisors to HPF who is conducting this research in fulfillment of a PhD. HPF, CG, BW, MF, AB and TC developed the physiotherapy intervention protocols. HPF will act as trial co-ordinator and was responsible for drafting this paper, although all authors provided comments on the draft. All authors have read and approved the final manuscript.

## Pre-publication history

The pre-publication history for this paper can be accessed here:


